# Coronary Physiology Across the Whole Spectrum of Ischemic Heart Disease

**DOI:** 10.3390/jcm15031313

**Published:** 2026-02-06

**Authors:** Ciro Pollio Benvenuto, Luigi Cappannoli, Andrea Viceré, Vincenzo Viccaro, Simona Todisco, Chiara Giuliana, Faisal Sharif, Domenico Galante

**Affiliations:** 1 Department of Cardiovascular Science, Università Cattolica del Sacro Cuore, Largo Agostino Gemelli 8, 00168 Rome, Italy; 2 St. Antonius Ziekenhuis, Koekoekslaan 1, 3435 CM Nieuwegein, The Netherlands; 3 Fondazione Policlinico Universitario Agostino Gemelli IRCCS, Largo Agostino Gemelli 8, 00168 Rome, Italy; 4 Department of Cardiology, Galway University Hospital, H91 YR71 Galway, Ireland; 5 Sharif Cardiovascular Research Group, University of Galway, H91 YR71 Galway, Ireland; 6 Center of Excellence of Cardiovascular Sciences, Ospedale Isola Tiberina-Gemelli Isola, 00186 Rome, Italy

**Keywords:** coronary physiology, Fractional Flow Reserve, Coronary Flow Reserve, Index of Microcirculatory Resistance, *#FullPhysiology*

## Abstract

Acute and Chronic Coronary Syndromes represent two major medical challenges and are the leading cause of cardiovascular mortality and morbidity. While Chronic Coronary Syndrome (CCS) can be defined as the whole group of structural and/or functional abnormalities involving coronary arteries before and after an acute event, Acute Coronary Syndrome (ACS) encompasses the condition of acute myocardial ischemia (with or without consequent myocardial injury and troponin release) due to dynamic mechanisms such as athero-thrombosis or vasospasm. Because of this complex interplay between structural and functional mechanisms arising from both the epicardial and microvascular compartments, a comprehensive approach to fully investigate the whole spectrum of coronary disease is therefore essential. To address this issue, the invasive functional assessment has evolved through the years, from a way to guide revascularization to a meticulous protocol for characterizing ischemia-leading mechanisms and stratifying prognosis both in ACS and CCS. However, coronary physiology remains underused in clinical practice, and multiple gaps in knowledge still exist; on top of this, there is increasing heterogeneity regarding how to perform functional assessment, with different protocols proposed by various centers. The aim of this review is to summarize the evidence in the field of coronary physiology, and to discuss how and when to use it at its best.

## 1. Introduction

The clinical spectrum of coronary artery disease spans from well-controlled chronic conditions to life-threatening acute events. Understanding the pathophysiological mechanisms leading to myocardial ischemia in both Acute and Chronic Coronary Syndromes is fundamental to improve patient care [1] (Central Illustration). For this purpose, it is essential to clarify the anatomy and physiology of the coronary circulation in order to understand the pathophysiology behind coronary artery disease.

### Coronary Anatomy and Physiology

Even if the coronary tree is divided into compartments—the epicardial and the microvascular ones—for didactic purposes, it should be seen as a continuum [2]. The most well-known and investigated compartment is the epicardial one, because it is macroscopically visible and can be studied via conventional angiography; it consists of vessels ranging from a few millimeters to about 500 μm. These are the conduit vessels, where—if no atherosclerosis is present—friction and resistance to flow are minimal, allowing for maximal driving pressure. The coronary circulation then continues with the pre-arterioles (diameters between 500 and 100 μm)—located in the extramural space and thus insensitive to myocardial metabolites—and with the arterioles (diameters under 100 μm)—located in the intramural space and responsive to endogenous metabolites like adenosine [3]. Pre-arterioles and arterioles are considered the “microcirculation”, not visible with current diagnostic tools. They act as resistance vessels, regulating coronary blood flow in response to oxygen demand: a mechanism called autoregulation [4]. Due to the resistance offered by pre-arterioles and arterioles, the downstream perfusion pressure drops to the levels found in the coronary sinus.

Multiple factors are involved in the control of their vascular tone, such as neuro-hormonal, mechanical, and metabolic factors, whereby the most potent endogenous vasodilator is adenosine, an end product of human metabolism. Adenosine acts directly on the smooth muscle cells surrounding the arteriolar wall; therefore, it is an endothelium-independent agent, and its use in clinical practice is not hindered by concomitant endothelial dysfunction. Overall, epicardial vessels account for 5–10% of coronary flow resistance (without atherosclerosis), while pre-arterioles and arterioles account for 20–30% and 40–60%, respectively [5].

It is also important to note that coronary flow is not homogeneous throughout the cardiac cycle: it is minimal during isovolumetric systole and even retrograde at the systolic peak, while it is maximal during diastole, particularly during ventricular suction [6]. Moreover, flow distribution is not uniform across myocardial layers: the subendocardial layers receive less blood flow compared to the subepicardial layers due to higher tension across the myocardial wall, making the endocardium more vulnerable to ischemia [7].

**Figure 1:**
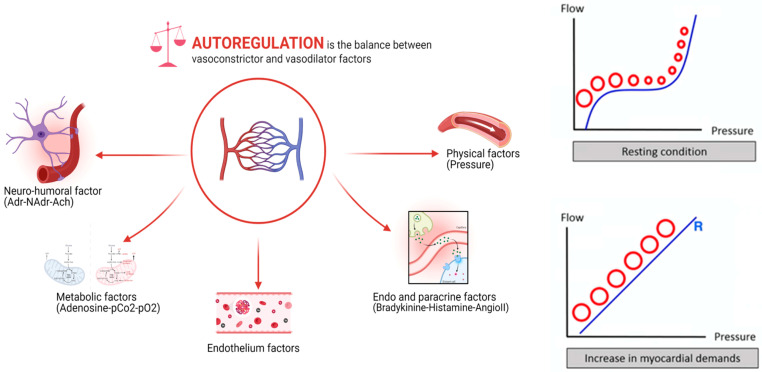
Autoregulation in coronary physiology is regulated by multiple factors control the vascular tone of pre-arterioles and arterioles, which maintain resting flow constant until the point of maximal vasodilatation, when flow is driven exclusively by the perfusion pressure. At that point, pressure and flow can be assumed to be equivalent.

One of the most important concepts in coronary physiology is the aforementioned autoregulation (Figure 1), namely the ability to maintain constant blood flow, even to the detriment of perfusion pressure [8]. Lance Gould demonstrated this phenomenon for the first time by progressively narrowing an epicardial artery in unsedated dogs: indeed, maximal coronary flow decreased only with more than 50% diameter reduction, while resting flow was preserved until 90% narrowing, a threshold beyond which even resting flow declined [9]. This means that increased resistance from epicardial atherosclerosis can be counterbalanced by reduced microvascular resistance, preserving resting flow and avoiding symptoms until exertion, during which microvascular compensatory vasodilation is finally exhausted [10]. In order to understand this phenomenon, it is necessary to acknowledge that the myocardium can meet an increased oxygen demand only by increasing oxygen delivery, and not by increasing its extraction, simply because oxygen extraction is already maximal at rest [5].

## 2. The Role of Functional Assessment in Chronic Coronary Syndrome

### 2.1. Guiding Revascularization

Since its birth, coronary physiology has empowered the decision-making process in patients with “stable” coronary artery disease [11]. Rather than relying solely on operators’ visual assessment, coronary physiology allows for a more accurate identification of ischemia-inducing lesions, thereby optimizing outcomes and reducing unnecessary interventions. In this regard, it has been proven that only flow-limiting lesions benefit from revascularization on top of medical therapy, and that angiography itself is not properly able to identify these lesions, overestimating their relevance and thus leading to unnecessary and potentially harmful procedures [12]. For this reason, current guidelines recommend (class I; level of evidence A) that the invasive functional assessment should be used to properly select patients requiring further interventions—especially if non-invasive ischemia tests are not available—and that angiography should be trusted only in the case of critical lesions (90% or more) [1,13]. Regarding the modalities to perform this assessment, multiple indices have been investigated and will be discussed in the following paragraphs.

#### 2.1.1. Fractional Flow Reserve (FFR)

Fractional Flow Reserve (FFR) is the gold standard for decision-making in “stable” coronary artery disease. It is defined as the ratio of distal coronary pressure (Pd) to aortic pressure (PA) during pharmacologically induced maximal hyperemia, and it should be considered—assuming the equivalence between flow and pressure in ideal conditions (e.g., hyperemia)—as the ratio between the actual flow and the maximal flow in the absence of epicardial disease [14]. It reflects the ischemic burden in the investigated vessel, and—although ischemia is a continuum—a binary cut-off of 0.80 is used in clinical practice to define the need for revascularization.

FFR was initially tested as a metric to safely defer PCI, as shown by the DEFER (deferral vs. performance of percutaneous coronary intervention of functionally non-significant coronary stenosis: 15-year follow-up of the DEFER) trial [15]. The DEFER study enrolled 325 patients and randomized them to revascularization or conservative management, according to an FFR value of 0.75. Five- and fifteen-year follow-up showed no difference in cardiac death, but a reduction in myocardial infarction (MI) in the deferred group due to a decrease in procedural MIs [15,16]. This paved the way for the use of FFR as a deferral tool.

Subsequently, the FAME (Fractional Flow Reserve versus Angiography for Multivessel Evaluation) study demonstrated that, in patients with multivessel disease, FFR-guided percutaneous coronary intervention (PCI) is associated with a lower incidence of death, myocardial infarction, and repeated revascularizations compared to angiography-guided PCI [17]. Following the FAME study, the FAME (Fractional Flow Reserve-Guided PCI versus Medical Therapy in Stable Coronary Disease) 2 trial compared FFR-guided PCI and optimal medical therapy (OMT) against OMT alone [18]. The study was stopped early due to a significant reduction in the rate of urgent revascularizations in the PCI group, further reinforcing the role of physiology in improving patient outcomes. Both FAME and FAME 2 trials used an FFR cut-off of 0.80 in order to improve sensitivity over specificity [14]. The results of both trials were consistent at the 5-year follow-up [19,20].

Finally, the FAME (Fractional Flow Reserve-Guided PCI as Compared with Coronary Bypass Surgery)3 trial randomized 1500 patients to FFR-guided PCI or coronary artery bypass grafting (CABG), showing the inferiority of the former approach in reducing the composite endpoint of death, MI, stroke or repeated revascularizations; this difference was mainly driven by higher rates of repeated revascularizations and MIs in the PCI group [21]. Recently, a 5-year follow-up of the FAME 3 trial was published, showing no difference in the composite outcome of death, myocardial infarction, or stroke [22]. Despite a still-higher rate of MIs in the FFR-guided PCI group, this approach proved to be a safe alternative to CABG when considering only death as a “hard” endpoint [23].

Based on the FAME trilogy’s results, these are the conclusions which can be drawn: by measuring FFR, a safe deferral for non-ischemic lesions is guaranteed, avoiding the “over-stenting” associated with the angiographic guidance and the subsequent risk of peri-procedural MIs, while, on the other hand, a benefit over medical therapy—mainly due to a lower rate of urgent revascularizations—is obtained for FFR-positive cases; on top of this, FFR-guided PCI can be considered as a relatively safe alternative to CABG, with no significant increase in the rate of the most critical endpoint—death—at the price of a higher incidence of MIs and repeated revascularizations. Taken together, these results demonstrate how FFR is the metric of choice in CCS patients.

#### 2.1.2. Alternatives to FFR

**Instantaneous wave-free ratio:** The instantaneous wave-free ratio (iFR) is a non-hyperemic, pressure-derived index that specifically targets a diastolic interval known as the *wave-free period*, during which microvascular resistance is assumed to be stable and minimal [24]. By exploiting this physiological window, iFR eliminates the need for vasodilator agents such as adenosine, thereby enhancing patients’ safety and saving costs and time [25]. The DEFINE-FLAIR [26] and iFR-SWEDEHEART [27] trials—each enrolling over 2000 patients and thus representing the largest studies in the field of coronary physiology—demonstrated the non-inferiority of iFR in comparison with FFR at one-year follow-up. Both trials used the composite primary endpoint, including all-cause mortality, myocardial infarction, and unplanned revascularization. As a result, the 2018 ESC/EACTS Guidelines on Myocardial Revascularization [28] and the 2019 ESC Guidelines on Chronic Coronary Syndromes [29] recommended both iFR and FFR for the assessment of intermediate coronary lesions. However, the long-term follow-up of the aforementioned studies has yielded mixed findings. While the iFR-SWEDEHEART trial confirmed no significant differences in outcomes—whether composite or individual—at five years [30], the DEFINE-FLAIR trial reported an unexpected increase in all-cause mortality in the iFR group, despite no differences in the rate of myocardial infarction or unplanned revascularization [31]. Furthermore, two meta-analyses have identified an excess in mortality—and in major adverse cardiovascular events (MACE), primarily driven by all-cause death—without a corresponding increase in the rate of myocardial infarctions or unplanned revascularizations [32,33]. Despite these controversial findings, the latest guidelines from the European Society of Cardiology (ESC) keep supporting iFR as an alternative to FFR [1]. Currently no definitive conclusions can be drawn, since the increase in mortality seems not to be linked to an excess in the rate of myocardial infarctions or revascularizations, and further studies are urgently required to clarify this issue. In routine clinical practice, it makes sense for most interventionalists to measure FFR in the presence of borderline iFR values, and then trust FFR.

**cFFR:** Contrast-derived Fractional Flow Reserve (cFFR) has emerged as a promising alternative to the traditional adenosine-induced FFR when assessing the functional significance of coronary artery stenoses [34]. By taking advantage of the transient and mild hyperemic effect elicited by intracoronary contrast medium injection [35], cFFR offers a simplified and cost-effective approach for epicardial assessment. Several studies have demonstrated the diagnostic accuracy of cFFR in comparison to standard FFR: the RINASCI study reported a strong correlation between cFFR and FFR values (r = 0.94, *p* < 0.001), with a cFFR cutoff of ≤0.83 yielding high sensitivity (85.7%) and specificity (96.1%) for predicting an FFR ≤ 0.80 [36]. Similarly, the MEMENTO-FFR study, encompassing over 1000 coronary lesions, found cFFR to be more accurate than resting Pd/PA and iFR in identifying functionally significant stenoses [37]. Finally, Johnson et al. compared resting Pd/PA ratio, iFR, and cFFR against adenosine-induced FFR in 763 patients: cFFR showed the best diagnostic performance (AUC 0.93) with an optimal cutoff of 0.83, outperforming both resting Pd/PA and iFR [38]. The study demonstrated that there is a physiological continuum from rest to the full, adenosine-induced hyperemia, suggesting that cFFR provides a practical, adenosine-free and mildly hyperemic solution. However, since hyperemia is submaximal with contrast medium, cFFR should complement—and not replace—standard FFR, particularly in borderline cases. In this regard, Leone at al. [36] proposed a hybrid approach: the functional relevance of a stenosis is considered to be positive when cFFR is ≤0.83 and negative when cFFR > 0.88, while the use of FFR is suggested for the remaining cases.

Importantly, the diagnostic performance of cFFR appears consistent across different contrast media types and volumes. A study by Nishi et al. concluded that neither contrast osmolality nor volume significantly affected the overall accuracy of cFFR, although they did influence sensitivity and specificity [39]. Given its ease of use and favorable safety profile, cFFR represents a good alternative to FFR [40], particularly in settings where adenosine administration is contraindicated or impractical. However, it should be acknowledged that cFFR has never been tested against FFR in randomized trials concerning clinical endpoints, so further studies are necessary to confirm its role as a valid alternative. Nevertheless, Leone et al. [41] demonstrated that even in cases of discordance between cFFR and FFR, the use of cFFR does not result in any difference in patients’ outcomes.

**Other Non-Hyperemic Pressure Ratios (NHPRs):** Besides iFR, several other Non-Hyperemic Pressure Ratios (NHPRs) have been proposed as adenosine-free alternatives for functional assessment [42]. These include the diastolic pressure ratio (dPR), the resting full-cycle ratio (RFR), and the diastolic hyperemia-free ratio (DFR). All of them rely on pressure ratios acquired during specific parts of the cardiac cycle under resting conditions, avoiding the need of pharmacologically induced hyperemia. Among these, RFR measures the lowest Pd/PA value across the entire cardiac cycle, while dPR and DFR are restricted to diastolic intervals.

Multiple studies have shown these NHPRs to have excellent correlation and near-equivalence with iFR [43,44], with diagnostic accuracy exceeding 80% when predicting FFR ≤ 0.80 [45]. For this reason, according to the 2024 ESC guidelines for CCS management, NHPRs may be considered as an alternative to FFR in patients with intermediate coronary stenoses [1].

Regardless of the adopted index, functional assessment of the epicardial compartment should be performed to evaluate the need for revascularization, especially in patients with intermediate stenosis, when angiography is not sufficiently informative, and when non-invasive ischemia tests are not available. The implementation of coronary physiology in the cath lab has been proven to lead to less revascularizations, without an increase in cardiovascular events and with a reduction in peri-procedural myocardial infarction and unnecessary procedures [12]: indeed, the deferral of negative lesions has been demonstrated to be safe, while positive ones should be referred for further interventions, on top of medical therapy, in order to improve symptoms and to prevent future ischemia-driven revascularizations.

#### 2.1.3. Pullback Pressure Gradient (PPG)

The functional impact of coronary artery disease (CAD) resulting from epicardial atherosclerosis is not always the same, since the pressure drop can be primarily focused around a stenosis or distributed along the entire vessel. These two patterns, focal and diffuse, both from anatomical and functional standpoints, represent the two extremes of a continuum of different modalities of flow impairment [46]. Unfortunately, conventional angiography is unable to properly assess the functional burden imposed by atherosclerosis, while a valuable aid is provided by the pullback maneuver during continuous hyperemia [47]. When the pullback maneuver is performed, the pressure drop is plotted against the vessel length, so that its spatial distribution can be properly appreciated, and the prevalent pattern of disease identified. However, while it is relatively easy to discern between predominantly focal and predominantly diffuse patterns of disease, the majority of patients display mixed features, making the interpretation of the pullback curve more challenging. To overcome the subjectivity and variability of the visual interpretation, a novel quantitative index, the Pullback Pressure Gradient (PPG), has been developed and investigated [48], showing that PPG is able to standardize pullback analysis and allow the assessment of CAD patterns, with an excellent inter- and intra-observer agreement [49]. PPG was initially validated with a motorized device performing the pullback maneuver at a speed of 1 mm/s during continuous hyperemia [47]. However, motorized pullback devices are cumbersome to use, not widely available, and may require longer intravenous adenosine infusions. Therefore, PPG was subsequently validated using manual pullback, which is more convenient for routine clinical practice since it takes from 20 to 30 s to perform a constant and slow manual pullback of the pressure wire [50].

Recently, the PPG Global registry [51] demonstrated that a high PPG value—so a focal pattern of disease—can predict optimal functional results after PCI (post-PCI FFR ≥ 0.88) more accurately than FFR alone; beside, PPG influenced treatment decisions in 14% of patients, since in case of diffuse disease other treatments (OMT or CABG) were preferred over PCI. Moreover, peri-procedural myocardial infarctions occurred more frequently in patients with lower PPG values (<0.62), probably due to the use of longer stents in smaller arteries, with a higher rate of post-dilations. Finally, effective angina relief is less frequent in patients with lower PPG values, further demonstrating how this metric can predict the likelihood of functionally suboptimal PCI. Thus, PPG represents a promising tool in the physiological stratification of CAD, helping physicians better tailor treatment [52].

### 2.2. PCI Optimization

While coronary physiology is a well-established tool to assess the need for revascularization, its systematic use following PCI remains limited, despite increasing evidence supporting its prognostic value [53]. A growing body of literature demonstrates that the angiographic success after PCI is not always associated with optimal physiological outcomes [54]. In this regard, suboptimal post-PCI functional results, defined by low FFR, are frequent and independently associated with adverse outcomes [55]. Several large studies, including FFR-SEARCH [56] and REPEAT-FFR [57], have shown that up to 50–60% of lesions treated with PCI may exhibit post-PCI FFR values ≤ 0.90, with up to 20% still in the ischemic range (FFR ≤ 0.80). This phenomenon is not rare, and significantly correlates with increased rates of target vessel failure (TVF) and target vessel revascularization (TVR): indeed, in the PROPHET-FFR registry [58], patients undergoing post-PCI FFR measurements and optimization achieved higher final FFR values and experienced fewer events, mainly because of a reduction in TVRs; conversely, the group managed solely by angiographic guidance showed a higher risk of cardiovascular events. These data support the concept that functional optimization—not just anatomical correction—should be the endpoint of PCI [59].

Despite angiographic success, suboptimal post-PCI FFR can arise from various causes [60]. Technical factors, such as pressure drift, can produce misleading results and must be excluded through proper equalization and curve assessment [61]. Physiological contributors include coronary tortuosity, with subsequent accordion phenomenon, or vasospasm, which requires the administration of nitrates as per standard fashion [62]. Other critical contributors can be stent-related factors, such as underexpansion or malapposition, or related to residual disease, either diffuse or focal [63]. To identify these issues, both intracoronary imaging and hyperemic pullback are recommended. The DOCTORS study [64] demonstrated that OCT-guided PCI leads to significantly higher post-PCI FFR values than angiography-guided PCI, largely by enabling correction of stent underexpansion. Similarly, the OxOPT-PCI trial [65] showed that impaired FFR (<0.90) often correlates with OCT-detected stent abnormalities, and optimization based on imaging criteria can improve final FFR. Similar results were also obtained in the IVUS substudy of the FFR-SEARCH registry [66]: in vessels with a post-PCI value below 0.85, stent underexpansion and malapposition were found in 74% and 22% of vessels, respectively, while relevant residual focal lesions were found proximally and distally to the stented segment in 29% and 30% of cases, respectively. These findings stress the need for a multimodal approach that integrates functional data with intravascular imaging [67]: the latter can be used to optimize stent implantation and correct stent-related issues, while the pullback maneuver can unmask residual diffuse disease not amenable to further interventions. However, post-PCI functional assessment is hampered by the iatrogenic risk associated with vessel rewiring with the pressure wire and the administration of adenosine in a different, more critical clinical scenario. To address this issue, pressure microcatheters have been developed, and it has recently been demonstrated that monorail pressure microcatheters, due to their ease of use, are associated with a greater number of post-PCI functional evaluations and, consequently, physiology-guided optimizations [68]. This led to higher final FFR values in comparison to conventional pressure wires and, consequently, to a tendency towards better clinical outcomes. Finally, in the same study, it was demonstrated how cFFR could be safely used for post-PCI assessment in order to avoid adenosine administration [68].

There is no consensus about a specific post-PCI FFR threshold for prognostication. Early work in the era of balloon angioplasty and bare-metal stents showed that patients with post-PCI FFR > 0.90 had significantly lower MACE rates than those with FFR < 0.90 [69]. In the drug-eluting stent (DES) era, similar thresholds remain valid. For example, Doh et al. [70] and Nam et al. [71] found that FFR values above 0.89 or 0.91 predict a reduced need for revascularization, and, in the DK Crush VII registry, FFR < 0.88 independently predicted TVF at three years [72]. However, recent evidence suggests that a vessel-specific cut-off must be considered: indeed, the left anterior descending artery (LAD) tends to yield lower post-PCI FFR values due to its anatomical course (downward position in relation to the aortic root) and the greater amount of supplied myocardial mass [73], while the left circumflex tends to display higher value due to its upward position and the relative hydrostatic effect [74,75]. Accordingly, evidence from the Post-PCI FFR Registry [76] and Collet et al.’s meta-analysis [77] suggests a vessel-specific interpretation of post-PCI optimal FFR cut-offs: 0.86 for the LAD and 0.90 for other vessels. Regardless of the adopted threshold, it is important to be aware that ischemia is a continuum and so is the relative FFR value.

Recent evidence also suggests that every incremental improvement in FFR confers prognostic benefit. Hwang et al.’s individual patients’ data meta-analysis involving over 5000 patients identified FFR values of 0.86 and 0.80 as optimal cutoffs for predicting TVF and the composite endpoint of cardiac death or target vessel myocardial infarction, respectively. Notably, for every 0.01 increase in final FFR, the risk of future adverse events decreased, reinforcing the importance of striving for the best functional result. Importantly, even when post-PCI FFR exceeds the ischemic threshold, intermediate results (e.g., 0.81–0.85) are associated with a fair risk of adverse outcomes and should prompt consideration of further optimization, particularly in high-risk patients [78].

In summary, the evidence clearly supports incorporating post-PCI physiological assessment into routine clinical practice. Despite the widespread reliance on angiography, physiology reveals a substantial proportion of suboptimal results with prognostic relevance. Post-PCI FFR—complemented by imaging and hyperemic pullback—allows identification and correction of residual ischemia, which in turn improves outcomes. An FFR value > 0.90 may represent the ideal target, with lower vessel-specific thresholds in selected cases. The contemporary interventional strategy should evolve toward a fully physiology-guided PCI paradigm, emphasizing not only lesion selection but also procedural and functional success [79].

### 2.3. Defining the Endotype in ANOCA/INOCA and Guiding Tailored Treatment

#### 2.3.1. Definition and Epidemiology of INOCA/ANOCA

Angina and/or ischemia with no obstructive coronary arteries (Angina and/or Ischemia with No Obstructive Coronary Arteries, ANOCA/INOCA) require, by definition, the presence of anginal symptoms (in the case of ANOCA) and the objective evidence of myocardial ischemia (in the case of INOCA) without relevant epicardial disease [80]. Initially, this was referred to “normal or near-normal coronary arteries”, but more recently the definition has broadened to encompass even cases with angiographic evidence of coronary lesions, provided that they are not functionally significant: this underlines the critical role of pressure-based functional assessment to rule out epicardial disease as the reason for myocardial ischemia and to rule in patients for further investigations [81].

About half of the patients undergoing coronary angiography for symptoms and/or signs of myocardial ischemia do not have angiographic findings that explain the clinical presentation. This condition is even more frequent in women, where more than 50% of angiographic studies reveal coronary arteries free (or nearly so) of atherosclerotic disease [82]. Despite this, ANOCA and INOCA remain underdiagnosed and undertreated, significantly impacting patients’ quality of life and leading to unnecessary, costly, and invasive diagnostic procedures. This underscores the importance of proper recognition and treatment of ANOCA/INOCA [83].

#### 2.3.2. Pathophysiology of INOCA/ANOCA

In order to understand how invasive physiology can lead to the identification of ischemia-leading mechanisms and how it can be used to start tailored treatment, it is essential to understand the pathophysiology behind ANOCA/INOCA. Angina and ischemia can arise from structural and/or functional alterations of small coronary vessels, a condition known as coronary microvascular dysfunction (CMD), or from vasomotility abnormalities such as epicardial spasm [84]. These different pathogenic mechanisms can occur simultaneously and in combination with epicardial atherosclerotic disease, but they will be discussed individually for educational purposes.

**Structural CMD:** This condition is determined by arteriolar and pre-arteriolar remodeling, with intimal hyperplasia and smooth muscle cell proliferation, and capillary rarefaction [85]. This leads to an “anatomical” increase in microvascular resistance, as if there is a stenosis within the microcirculation, and myocardial ischemia is hence caused by the inability to lower microvascular resistance to match the increased oxygen demand (e.g., physical exercise). Hypertension and diabetes—common macro- and micro-vascular disrupting factors—are often associated with this condition [86].

**Functional CMD:** This condition, often seen in women, is characterized by the inability to increase coronary blood flow to meet oxygen demand, without concomitant evidence of relevant epicardial disease or structural abnormalities in the microcirculation: this means that this abnormality is not determined by a pathological increase in the resistance to blood flow, neither in the epicardial vessels nor in the small ones, but it is mainly due to increased resting flow [87]. This can be seen in patients with non-coronary disease, such as hypertrophic heart disease in the presence of long-lasting, poorly controlled hypertension, or even in the absence of heart disease, like in patients with anemia and compensatory increased resting flow. However, after the exclusion of potential confounding factors, the reason for ischemia should be seen in functional abnormalities leading to altered flow at rest and impaired coronary blood flow distribution within the myocardial layers [88].

**Vasospasm:** Spasm is defined as an abrupt vessel caliber reduction due to the contraction of smooth muscle cells. Although only epicardial spasm is visible by means of conventional angiography, the occurrence of microvascular spasm is also possible, as often seen in the presence of slow or sluggish flow [89]. This abnormal response can be triggered by intracoronary acetylcholine administration. Acetylcholine—an endogenous mediator—stimulates both endothelium-mediated (via nitric oxide) vasodilation and, on the other hand, smooth muscle cell contraction, which leads to vasoconstriction. Thus, endothelial dysfunction is a *conditio sine qua non* for any vasomotility disorders: indeed, when the vasoconstrictor stimulus on smooth muscle cells is not compensated by the endothelium-mediated vasodilation, a spasm can be evoked [90]. Another possible explanation could be smooth muscle cells hypereactivity, and this kind of individual predisposition often results in multivessel spasms involving other vascular beds as well (e.g., radial artery).

#### 2.3.3. Invasive Assessment in Patients with Suspected ANOCA and/or INOCA

After ruling out significant epicardial disease (or even in the presence of it), a comprehensive assessment of microvascular function and vasomotility disorders should be done with adenosine-based and acetylcholine-based investigations [91]. In this regard, adenosine must be administered via the venous system (preferably via a central vein) in order to reach steady-state hyperemia, and even if intracoronary administration is possible to measure FFR, the extremely rapid wash-out of the drug makes it impossible to perform the pullback maneuver or the microvascular assessment. For the same reason, even if the hyperemia induced by intracoronary papaverine can last up to 30 s, this time frame could still be insufficient for a proper microvascular assessment.

Collateral effects of intravenous adenosine are usually mild and transient, with an almost immediate resolution after discontinuation of the administration, and include dyspnea (rarely associated with bronchospasm), chest discomfort or chest pain, headache or “lightness” in the head, and flushing; bradycardia and atrio-ventricular block are more common with intracoronary administration.

When performing microvascular assessment, before at rest and then during steady-state hyperemia induced by intravenous adenosine, three injections of room-temperature saline are done to compute the *mean transit time* (*Tmn*) from thermodilution curves. This method is referred to as the bolus thermodilution technique, and it requires thermistor-equipped pressure wires and dedicated software. From this analysis, the Coronary Flow Reserve (CFR) and the Index of Microcirculatory Resistance (IMR) can be derived, respectively, as the ratio between resting and hyperemic *Tmn*, and as the product of distal pressure and *Tmn*, both recorded during steady-state hyperemia. CFR reflects the real ability to increase coronary blood flow to match oxygen demand, while IMR reflects the microvascular resistance to flow under hyperemia, when resistance should be minimal. It is important to remember that small vessels are responsible for autoregulation, since they act as “resistance” vessels, so that IMR—when pathologically increased—corresponds to the inability to lower the microvascular tone enough to meet oxygen demand. While CMD is defined by a low CFR value (<2.5), IMR discerns between the structural endotype, characterized by an anatomical increase in microvascular resistance (high IMR), and the functional endotype, defined by low CFR and preserved IMR [87].

**Figure 2:**
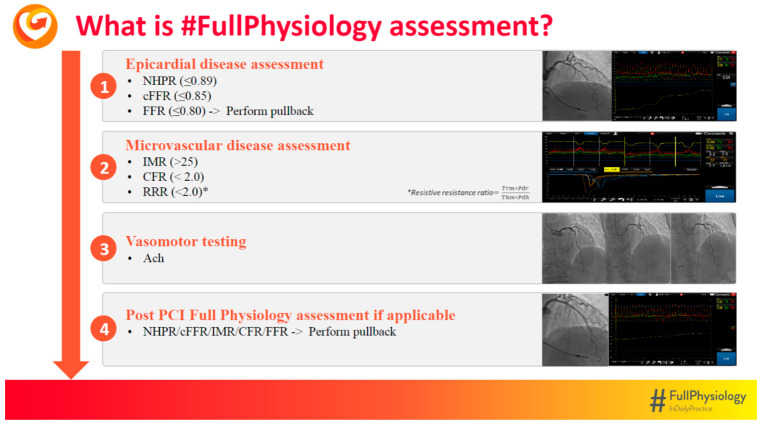
Showing the *#FullPhysiology* algorithm: 1. The first step is devoted to epicardial assessment: hyperemic metrics (FFR and/or cFFR) are strongly recommended over the NHPRs. 2. The second step involves the microvascular assessment via bolus thermodilution. 3. Acetylcholine provocation test is then done as the third part of the protocol. 4. Finally, if PCI is performed, the epicardial assessment should be repeated to check for the functional result.

Acetylcholine is administered via incremental intracoronary boluses to check for spasm, and can cause bradycardia and atrio-ventricular block, but also atrial fibrillation. These adverse effects are usually self-limiting, but they last longer than with adenosine because acetylcholine’s half-life is longer. Ventricular arrhythmias are extremely rare, but can occur. Epicardial and microvascular spasms are diagnosed following COVADIS criteria [92]: in both cases, the recurrence of angina—the typical chest pain referred by the patient—and the appearance of ECG ischemic changes are seen, but only epicardial spasm is confirmed by 90% or more vessel caliber reduction. A strict adherence to COVADIS criteria makes epicardial and microvascular spasm mutually exclusive, but they are not. As a matter of fact, under incremental doses of intracoronary acetylcholine, the appearance of slow flow—a clue of microvascular spasm—often comes before the epicardial spasm seen at higher acetylcholine doses. It should be acknowledged that all the aforementioned conditions can coexist, and that functionally significant epicardial disease can also be present, highlighting the importance of a comprehensive invasive functional assessment. In this regard, current European guidelines on CCS recommend the use of invasive functional testing to confirm (or exclude) the diagnosis of obstructive CAD and/or INOCA/ANOCA, especially in patients with no evidence of obstructive disease, but persistently symptomatic despite medical treatment, in order to identify potentially treatable endotypes and to improve symptoms and quality of life (class I; level of evidence B). Recently, the *#FullPhysiology* algorithm (Figure 2) was proposed as the ideal protocol to assess the entire coronary circulation and will be further discussed in the following paragraph [93].

### 2.4. How to Do Coronary Physiology in CCS: The #FullPhysiology Protocol

Given the pivotal role of coronary physiology in CCS, a stepwise approach, called *#FullPhysiology* protocol, has been proposed to perform a comprehensive assessment, and it has been demonstrated to be the most cost-effective strategy to improve quality of life and prognosis in patients with symptoms and/or signs of ischemia [94]. This multistep approach requires thermistor-equipped pressure wires, a dedicated software for online analysis, and the administration of vasoactive drugs. It has been divided into four sections, each one concerning a different feature of coronary artery disease.

The first part concerns the epicardial assessment. A hyperaemic index like FFR should be preferred over NHPRs, and cFFR should be reserved in cases in which adenosine is contraindicated. Finally, the macrovascular assessment should be completed with a manual pullback done under steady-state hyperemia. In this way, FFR is used to identify and stratify ischemic patients, and the PPG to choose the best way to manage positive cases, reserving PCI for mostly focal lesions. Even if the pullback can be done with NHPRs, such as iFR or RFR, it is recommended to perform the pullback under steady-state hyperemia, especially when multiple, tandem lesions are present: in this case, hyperemia can unmask the functional impact of minor lesions or concomitant diffuse disease.

The second part is related to microvascular assessment. Bolus thermodilution is used to compute *mean transit times* (*Tmn*)—both at rest and during hyperemia—from the thermodilution curves, and CFR and IMR are computed as previously described. While CFR is used to identify patients with CMD (if no functionally significant epicardial disease is present), IMR is used to characterize CMD as structural or functional.

The third part is devoted to vasomotility, investigated through acetylcholine administration via incremental intracoronary boluses, 20 mcg, 50 mcg, and then 100 mcg, according to the *#FullPhysiology* protocol. Acetylcholine is a vasoactive agent with mixed properties, eliciting a vasodilator effect (via nitric oxide) on endothelial cells and a vasoconstrictor effect on smooth muscle cells. Thus, in the presence of endothelial dysfunction and/or smooth muscle cells hypereactivity, spasm can be triggered. Epicardial and microvascular spasm are defined according to COVADIS criteria. It is suggested to leave the pressure wire in situ for back-up.

Finally, the last step concerns the post-PCI assessment. In FFR-positive and mainly focal lesions, PCI is the preferred choice for revascularization, but the functional assessment should be repeated in order to identify suboptimal results at risk of further revascularization driven by residual ischemia. It is important to repeat not only the FFR measurement, but also the pullback to understand if the residual pressure drop is stent-related, requiring further maneuver like post-dilatation, or due to residual disease outside the stented segment. Imaging is strongly recommended in case of suboptimal results.

**Figure 3:**
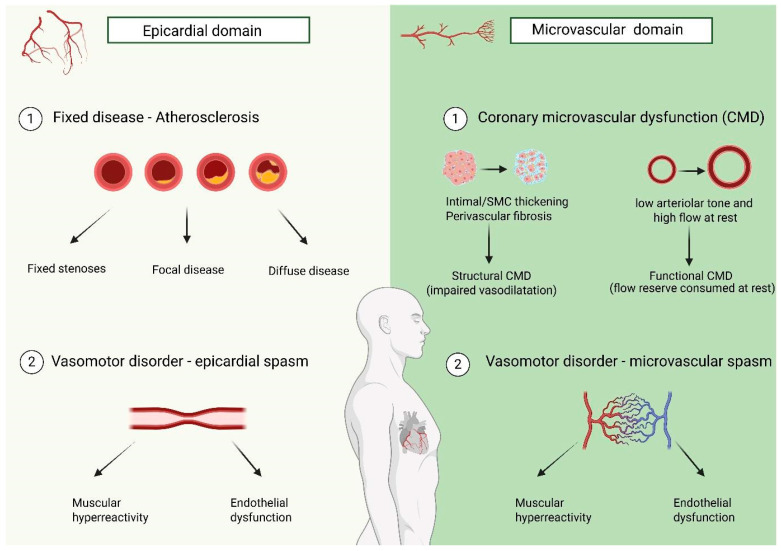
Showing the multiple mechanism leading to myocardial ischemia.

Via this multistep approach, the coronary circulation can be assessed as a whole, and ischemic mechanisms can be properly identified, even if multiple ones are coexisting (Figure 3). For example, patients with functionally significant atherosclerotic disease and concomitant microvascular or vasomotility disorders can already be informed that symptoms may persist even after successful PCI, and that tailored medical therapy is necessary to relieve them. Moreover, the precise characterization of endotypes is essential to tailor treatment: for example, vasospasm responds to calcium channel blockers and nitrates, whereas beta-blockers are contraindicated since they can provoke and worsen spasm. Furthermore, via this method, it is possible to rule out every possible coronary abnormality and to reassure patients that their complaints are of non-coronary origin [95].

However, while the epicardial assessment via FFR (or iFR) is strongly recommended by the ESC guidelines (class I; level of evidence A) and the comprehensive assessment of the entire coronary circulation to precisely identify the endotype is also strongly advised (class I; level of evidence B), the use of post-PCI functional assessment (with the implementation of PPG) is mainly endorsed by consensus documents, with increasing evidence from registries and observational studies, but no definitive findings from randomized clinical trials.

## 3. The Role of Functional Assessment in Acute Coronary Syndrome

### 3.1. To Define MINOCA

Myocardial infarction with non-obstructive coronary arteries (MINOCA) is a heterogeneous clinical syndrome affecting approximately 5–15% of acute myocardial infarction cases and is disproportionately represented in younger women. Unlike plaque-related myocardial infarctions, MINOCA encompasses a spectrum that includes coronary spasm, spontaneous coronary artery dissection, coronary embolism, and Takotsubo syndrome. Given this diversity, a pathophysiology-driven diagnostic approach is essential [96]. While angiography effectively rules out obstructive disease, it provides limited functional insight. To address this issue, a comprehensive protocol should encompass the use of intracoronary imaging and physiology—and eventually ventriculography—and it should continue outside the cath lab with CMR to identify possible mimickers such as myocarditis [97]. With regard to this, invasive physiology—mainly represented in this setting by the provocative test with acetylcholine—plays a pivotal role since it can unmask epicardial or microvascular spasm [98]. Montone et al. proved that patients with a positive test are at higher risk of major adverse cardiovascular events, and that acetylcholine administration is safe, with only transient and mild complications overall [99].

Despite these crucial findings, invasive physiology in MINOCA is underused and not yet included in standard guidelines. Barriers include procedural time, concerns about possible complications, and limited operator experience. Recently, the PROMISE trial showed that comprehensive diagnostic workup and consequent stratified treatment are able to improve symptoms at follow-up in comparison with standard care [100]. Despite the lack of statistical power to detect a valuable difference in clinical outcomes, this study is the first one showing how precision medicine can be safely applied in this setting, with positive implications for patients’ quality of life. According to the PROMISE study, invasive physiology was used in combination with intravascular imaging and cardiac MRI to identify the underlying etiology. While invasive physiology and intravascular imaging can unmask coronary-related abnormalities, cardiac MRI can distinguish between ischemic and inflammatory or stress-related injuries. The HARP-MINOCA study showed that OCT and MRI can identify the subtended mechanism in approximately 85% of cases, underscoring the value of this comprehensive strategy [101].

In conclusion, invasive physiological assessment in MINOCA offers crucial mechanistic insight and therapeutic guidance. Although evidence is not conclusive, the safety and diagnostic yield of this investigation support integrating physiology into routine evaluation of MINOCA patients.

### 3.2. To Stratify Prognosis After STEMI

In recent years, microvascular injury has been increasingly recognized as a key contributor to adverse outcomes in STEMI patients [102]. Multiple mechanisms—such as distal embolization of thrombotic debris, reperfusion-induced mechanical damage to small vessels, and neuro-hormonal activation impairing microvascular vasodilation—can compromise myocardial perfusion even after angiographically successful revascularization [103]. Traditionally, the Thrombolysis In Myocardial Infarction (TIMI) Flow Grade and myocardial blushing grade have been used as visual surrogates of perfusion quality, allowing gross detection of flow impairment. However, these scales lack sensitivity in identifying more subtle or “subclinical” microvascular dysfunction, which is still associated with worse prognosis.

To improve risk stratification, invasive physiology metrics have been applied at the end of primary percutaneous coronary intervention (pPCI). In particular, the Index of Microcirculatory Resistance (IMR)—measured using bolus thermodilution during maximal hyperemia—has emerged as the most robust invasive metric [104]. Early work by Fearon et al. in 2008 established that IMR measured immediately post-PCI independently predicted death, heart failure, and rehospitalization, with a threshold of 40 units identifying high-risk patients [105]. Complementary research from the Oxford Acute Myocardial Infarction (OxAMI) registry highlighted IMR’s ability to predict early complications [106]. In over 200 STEMI patients, IMR—measured post-pPCI—accurately stratified patients with regard to cardiovascular events such as heart failure. These observations underline the prognostic value of early recognition of microvascular damage.

Pathophysiological correlation with imaging has reinforced the validity of invasive measures. Studies using cardiac MRI consistently show that elevated IMR correlates with indicators of microvascular injury—such as infarct size, microvascular obstruction, and intramyocardial hemorrhage—validating its mechanistic relevance [107]. On top of this, the prognostic value of these findings has been subsequently validated and refined: in a pooled analysis, an IMR > 40 units was associated with larger infarct size, microvascular obstruction, and intramyocardial hemorrhage on cardiac magnetic resonance (CMR) imaging, all linked to worse outcomes [108]. Notably, Coronary Flow Reserve (CFR) did not independently predict long-term mortality in this cohort, solidifying IMR’s unique prognostic value.

Despite its strong prognostic performance, routine IMR measurement remains limited due to procedural complexity, costs, and its intrinsic iatrogenic risk. To address these limitations, angiography-derived methods have been developed. These rely on computational fluid dynamics applied to three-dimensional angiographic reconstructions in combination with surrogate markers of flow (contrast medium’s transit time), avoiding the need for thermodilution or hyperemia [109]. De Maria et al. demonstrated that an angiography-derived IMR (AngioIMR) correlates closely with thermodilution-derived IMR, and successfully stratifies microvascular dysfunction in STEMI [110]. More recently, Scarsini et al. validated this index as a tool to define the risk of early discharge: in this study, patients with lower AngioIMR were more likely to have uncomplicated clinical courses, suggesting practical utility in acute decision-making [111]. Finally, Pollio Benvenuto et al. showed the ability of AngioIMR to predict relevant outcomes—such as death and hospitalizations for heart failure—in the long term, over a 5-year follow-up [112]. However, due to the retrospective nature of these studies, further prospective studies, adequately powered and designed to precisely test a defined protocol of acquisition for AngioIMR, are necessary.

In summary, coronary physiology has established itself as a powerful tool for prognostic stratification in STEMI. IMR measured immediately after pPCI independently predicts mid- and long-term outcomes, including cardiac death and heart failure, while angiography-derived IMR offers a promising, less invasive alternative. The routine adoption of post-pPCI physiology will enable more accurate, personalized risk stratification and guide adjunctive therapies targeting the microcirculation.

## 4. Conclusions

Coronary physiology has evolved from a simple decisional tool to a cornerstone in precision medicine. From guiding revascularization in stable CAD to unmasking residual ischemia post-PCI, stratifying risk in STEMI, and elucidating mechanisms in ischemic syndromes with non-obstructive disease, physiological metrics offer unmatched insight into coronary pathophysiology. Despite robust evidence, their underuse persists. Standardizing their application across clinical scenarios—both chronic and acute—can refine treatment, reduce unnecessary interventions, and improve outcomes. A paradigm shift toward a physiology-first approach is no longer aspirational but essential for modern coronary care.

## Data Availability

No new data were created or analyzed in this study.

## References

[1] Vrints C., Andreotti F., Koskinas K.C., Rossello X., Adamo M., Ainslie J., Banning A.P., Budaj A., Buechel R.R., Chiariello G.A. (2024). ESC Scientific Document Group. 2024 ESC Guidelines for the management of chronic coronary syndromes. Eur. Heart J..

[2] Pupita G., Maseri A., Kaski J.C., Galassi A.R., Gavrielides S., Davies G., Crea F. (1990). Myocardial ischemia caused by distal coronary-artery constriction in stable angina pectoris. N. Engl. J. Med..

[3] Camici P.G., Crea F. (2007). Coronary microvascular dysfunction. N. Engl. J. Med..

[4] Mahendiran T., Collet C., De Bruyne B. (2024). Coronary-Artery Autoregulation with Increasing Stenosis. N. Engl. J. Med..

[5] Crea F., Lanza G.A., Camici P.G. (2014). Coronary Microvascular Dysfunction.

[6] Davies J.E., Whinnett Z.I., Francis D.P., Manisty C.H., Aguado-Sierra J., Willson K., Foale R.A., Malik I.S., Hughes A.D., Parker K.H. (2006). Evidence of a dominant backward-propagating “suction” wave responsible for diastolic coronary filling in humans, attenuated in left ventricular hypertrophy. Circulation.

[7] Johnson N.P., Gould K.L. (2025). Subendocardial Ischemia: Does CMD Really Exist?. Cardiovasc. Revasc. Med..

[8] Gould K.L. (1978). Pressure-flow characteristics of coronary stenoses in unsedated dogs at rest and during coronary vasodilation. Circ. Res..

[9] Gould K.L., Lipscomb K., Hamilton G.W. (1974). Physiologic basis for assessing critical coronary stenosis. Instantaneous flow response and regional distribution during coronary hyperemia as measures of coronary flow reserve. Am. J. Cardiol..

[10] Gould K.L., Lipscomb K. (1974). Effects of coronary stenoses on coronary flow reserve and resistance. Am. J. Cardiol..

[11] Park S.J., Ahn J.M. (2020). How I became an FFR believer. EuroIntervention.

[12] Mangiacapra F., Paolucci L., De Bruyne B., Rioufol G., Hahn J.Y., Chen S.L., Koo B.K., Tonino P.A.L., van ’t Veer M., Motreff P. (2025). Physiology and Revascularization for Myocardial Endpoints (PRIME) Collaboration. Fractional flow reserve vs angiography to guide percutaneous coronary intervention: An individual patient data meta-analysis. Eur. Heart J..

[13] Galante D., La Vecchia G., Leone A.M., Crea F. (2025). What has changed in the management of chronic ischaemic heart disease? The new European Society of Cardiology Guidelines 2024. Eur. Heart J. Suppl..

[14] Pijls N.H., De Bruyne B., Peels K., Van Der Voort P.H., Bonnier H.J., Bartunek J., Koolen J.J. (1996). Measurement of fractional flow reserve to assess the functional severity of coronary-artery stenoses. N. Engl. J. Med..

[15] Zimmermann F.M., Ferrara A., Johnson N.P., van Nunen L.X., Escaned J., Albertsson P., Erbel R., Legrand V., Gwon H.C., Remkes W.S. (2015). Deferral vs. performance of percutaneous coronary intervention of functionally non-significant coronary stenosis: 15-year follow-up of the DEFER trial. Eur. Heart J..

[16] Pijls N.H., van Schaardenburgh P., Manoharan G., Boersma E., Bech J.W., van’t Veer M., Bär F., Hoorntje J., Koolen J., Wijns W. (2007). Percutaneous coronary intervention of functionally nonsignificant stenosis: 5-year follow-up of the DEFER Study. J. Am. Coll. Cardiol..

[17] Tonino P.A., De Bruyne B., Pijls N.H., Siebert U., Ikeno F., van’ t Veer M., Klauss V., Manoharan G., Engstrøm T., Oldroyd K.G. (2009). FAME Study Investigators. Fractional flow reserve versus angiography for guiding percutaneous coronary intervention. N. Engl. J. Med..

[18] De Bruyne B., Pijls N.H., Kalesan B., Barbato E., Tonino P.A., Piroth Z., Jagic N., Möbius-Winkler S., Rioufol G., Witt N. (2012). FAME 2 Trial Investigators. Fractional flow reserve-guided PCI versus medical therapy in stable coronary disease. N. Engl. J. Med..

[19] van Nunen L.X., Zimmermann F.M., Tonino P.A., Barbato E., Baumbach A., Engstrøm T., Klauss V., MacCarthy P.A., Manoharan G., Oldroyd K.G. (2015). FAME Study Investigators. Fractional flow reserve versus angiography for guidance of PCI in patients with multivessel coronary artery disease (FAME): 5-year follow-up of a randomised controlled trial. Lancet.

[20] Xaplanteris P., Fournier S., Pijls N.H.J., Fearon W.F., Barbato E., Tonino P.A.L., Engstrøm T., Kääb S., Dambrink J.H., Rioufol G. (2018). FAME 2 Investigators. Five-Year Outcomes with PCI Guided by Fractional Flow Reserve. N. Engl. J. Med..

[21] Fearon W.F., Zimmermann F.M., De Bruyne B., Piroth Z., van Straten A.H.M., Szekely L., Davidavičius G., Kalinauskas G., Mansour S., Kharbanda R. (2022). Fractional Flow Reserve-Guided PCI as Compared with Coronary Bypass Surgery. N. Engl. J. Med..

[22] Fearon W.F., Zimmermann F.M., Ding V.Y., Takahashi K., Piroth Z., van Straten A.H.M., Szekely L., Davidavičius G., Kalinauskas G., Mansour S. (2025). Outcomes after fractional flow reserve-guided percutaneous coronary intervention versus coronary artery bypass grafting (FAME 3): 5-year follow-up of a multicentre, open-label, randomised trial. Lancet.

[23] Leone A.M., Vergallo R. (2025). Weekly Journal Scan: The five-year follow-up of FAME 3 keeps open the never-ending debate about coronary artery bypass grafting vs percutaneous coronary intervention in patients with three-vessel disease. Eur. Heart J..

[24] Sen S., Escaned J., Malik I.S., Mikhail G.W., Foale R.A., Mila R., Tarkin J., Petraco R., Broyd C., Jabbour R. (2012). Development and validation of a new adenosine-independent index of stenosis severity from coronary wave-intensity analysis: Results of the ADVISE (ADenosine Vasodilator Independent Stenosis Evaluation) study. J. Am. Coll. Cardiol..

[25] de Waard G.A., Di Mario C., Lerman A., Serruys P.W., van Royen N. (2017). Instantaneous wave-free ratio to guide coronary revascularisation: Physiological framework, validation and differences from fractional flow reserve. EuroIntervention.

[26] Davies J.E., Sen S., Dehbi H.M., Al-Lamee R., Petraco R., Nijjer S.S., Bhindi R., Lehman S.J., Walters D., Sapontis J. (2017). Use of the Instantaneous Wave-free Ratio or Fractional Flow Reserve in PCI. N. Engl. J. Med..

[27] Götberg M., Christiansen E.H., Gudmundsdottir I.J., Sandhall L., Danielewicz M., Jakobsen L., Olsson S.E., Öhagen P., Olsson H., Omerovic E. (2017). Investigators. Instantaneous Wave-free Ratio versus Fractional Flow Reserve to Guide PCI. N. Engl. J. Med..

[28] Neumann F.J., Sousa-Uva M., Ahlsson A., Alfonso F., Banning A.P., Benedetto U., Byrne R.A., Collet J.P., Falk V., Head S.J. (2019). ESC Scientific Document Group. 2018 ESC/EACTS Guidelines on myocardial revascularization. Eur. Heart J..

[29] Knuuti J., Wijns W., Saraste A., Capodanno D., Barbato E., Funck-Brentano C., Prescott E., Storey R.F., Deaton C., Cuisset T. (2020). ESC Scientific Document Group. 2019 ESC Guidelines for the diagnosis and management of chronic coronary syndromes. Eur. Heart J..

[30] Götberg M., Berntorp K., Rylance R., Christiansen E.H., Yndigegn T., Gudmundsdottir I.J., Koul S., Sandhall L., Danielewicz M., Jakobsen L. (2022). 5-Year Outcomes of PCI Guided by Measurement of Instantaneous Wave-Free Ratio Versus Fractional Flow Reserve. J. Am. Coll. Cardiol..

[31] Escaned J., Travieso A., Dehbi H.M., Nijjer S.S., Sen S., Petraco R., Patel M., Serruys P.W., Davies J., DEFINE FLAIR Investigators (2025). Coronary Revascularization Guided with Fractional Flow Reserve or Instantaneous Wave-Free Ratio: A 5-Year Follow-Up of the DEFINE FLAIR Randomized Clinical Trial. JAMA Cardiol..

[32] Berry C., McClure J.D., Oldroyd K.G. (2017). Meta-Analysis of Death and Myocardial Infarction in the DEFINE-FLAIR and iFR-SWEDEHEART Trials. Circulation.

[33] Eftekhari A., Holck E.N., Westra J., Olsen N.T., Bruun N.H., Jensen L.O., Engstrøm T., Christiansen E.H. (2023). Instantaneous wave free ratio vs. fractional flow reserve and 5-year mortality: iFR SWEDEHEART and DEFINE FLAIR. Eur. Heart J..

[34] Leone A.M., Lassandro Pepe F., Arioti M., Crea F. (2018). Contrast Fractional Flow Reserve (cFFR): A pragmatic response to the call for simplification of invasive functional assessment. Int. J. Cardiol..

[35] Galante D., Migliaro S., Di Giusto F., Anastasia G., Petrolati E., Vicerè A., Zimbardo G., Cialdella P., Romagnoli E., Aurigemma C. (2024). Age and Vasodilator Response to Different Hyperemic Agents: Adenosine versus Contrast Medium. Rev. Cardiovasc. Med..

[36] Leone A.M., Scalone G., De Maria G., Tagliaferro F., Gardi A., Clemente F., Basile E., Cialdella P., De Caterina A., Porto I. (2015). Efficacy of Contrast Medium Induced Pd/Pa Ratio in Predicting Functional Significance of Intermediate Coronary Artery Stenosis Assessed by Fractional Flow Reserve: Insights from the RINASCI Study. EuroIntervention.

[37] Leone A.M., Martin-Reyes R., Baptista S.B., Amabile N., Raposo L., Franco Pelaez J.A., Trani C., Cialdella P., Basile E., Zimbardo G. (2016). The Multi-center Evaluation of the Accuracy of the Contrast MEdiumINducedPd/Pa RaTiO in Predicting FFR (MEMENTO-FFR) Study. EuroIntervention.

[38] Johnson N.P., Jeremias A., Zimmermann F.M., Adjedj J., Witt N., Hennigan B., Koo B.K., Maehara A., Matsumura M., Barbato E. (2016). Continuum of Vasodilator Stress from Rest to Contrast Medium to Adenosine Hyperemia for Fractional Flow Reserve Assessment. JACC Cardiovasc. Interv..

[39] Nishi T., Johnson N.P., De Bruyne B., Berry C., Gould K.L., Jeremias A., Oldroyd K.G., Kobayashi Y., Choi D.H., Pijls N.H.J. (2017). CONTRAST Study Investigators. Influence of Contrast Media Dose and Osmolality on the Diagnostic Performance of Contrast Fractional Flow Reserve. Circ. Cardiovasc. Interv..

[40] Leone A.M., Campo G., Gallo F., Pavasini R., Basile E., D’Amario D., Tebaldi M., Biscaglia S., Maietti E., Trani C. (2020). Adenosine-Free Indexes vs. Fractional Flow Reserve for Functional Assessment of Coronary Stenoses: Systematic Review and Meta-Analysis. Int. J. Cardiol..

[41] Leone A.M., Arioti M., Cialdella P., Vergallo R., Zimbardo G., Migliaro S., Anastasia G., Di Giusto F., Galante D., Basile E. (2021). Prognostic impact of FFR/contrast FFR discordance. Int. J. Cardiol..

[42] De Maria G.L., Garcia-Garcia H.M., Scarsini R., Hideo-Kajita A., Gonzalo López N., Leone A.M., Sarno G., Daemen J., Shlofmitz E., Jeremias A. (2020). Novel Indices of Coronary Physiology: Do We Need Alternatives to Fractional Flow Reserve?. Circ. Cardiovasc. Interv..

[43] Svanerud J., Ahn J.M., Jeremias A., van ’t Veer M., Gore A., Maehara A., Crowley A., Pijls N.H.J., De Bruyne B., Johnson N.P. (2018). Validation of a novel non-hyperaemic index of coronary artery stenosis severity: The Resting Full-cycle Ratio (VALIDATE RFR) study. EuroIntervention.

[44] Johnson N.P., Li W., Chen X., Hennigan B., Watkins S., Berry C., Fearon W.F., Oldroyd K.G. (2019). Diastolic pressure ratio: New approach and validation vs. the instantaneous wave-free ratio. Eur. Heart J..

[45] Vira A., Balanescu D.V., George J.A., Dixon S.R., Hanson I.D., Safian R.D. (2024). Diagnostic Performance of Diastolic Hyperemia-Free Ratio Compared with Invasive Fractional Flow Reserve for Evaluation of Coronary Artery Disease. Am. J. Cardiol..

[46] Scarsini R., Fezzi S., Leone A.M., De Maria G.L., Pighi M., Marcoli M., Tavella D., Pesarini G., Banning A.P., Barbato E. (2022). Functional Patterns of Coronary Disease: Diffuse, Focal, and Serial Lesions. JACC Cardiovasc. Interv..

[47] Collet C., Sonck J., Vandeloo B., Mizukami T., Roosens B., Lochy S., Argacha J.F., Schoors D., Colaiori I., Di Gioia G. (2019). Measurement of Hyperemic Pullback Pressure Gradients to Characterize Patterns of Coronary Atherosclerosis. J. Am. Coll. Cardiol..

[48] Munhoz D., Collet C., Mizukami T., Yong A., Leone A.M., Eftekhari A., Ko B., da Costa B.R., Berry C., Collison D. (2023). Rationale and design of the pullback pressure gradient (PPG) global registry. Am. Heart J..

[49] Carvalho P.E.P., Collet C., De Bruyne B., Munhoz D., Sonck J., Sara J.D.S., Strepkos D., Mutlu D., Alexandrou M., Ser O.S. (2025). The Pullback Pressure Gradient: A Physiologic Index to Differentiate Focal from Diffuse Coronary Artery Disease. JACC Adv..

[50] Sonck J., Mizukami T., Johnson N.P., Nagumo S., Gallinoro E., Candreva A., Mileva N., Munhoz D., Shinke T., Svanerud J. (2022). Development, validation, and reproducibility of the pullback pressure gradient (PPG) derived from manual fractional flow reserve pullbacks. Catheter. Cardiovasc. Interv..

[51] Collet C., Munhoz D., Mizukami T., Sonck J., Matsuo H., Shinke T., Ando H., Ko B., Biscaglia S., Rivero F. (2024). Influence of Pathophysiologic Patterns of Coronary Artery Disease on Immediate Percutaneous Coronary Intervention Outcomes. Circulation.

[52] Kechichian A., Mizukami T., Malhotra G., Spratt J.C., Ikeda K., Corradetti S., Munhoz D., Sakai K., Sonck J., Wyffels E. (2025). Pullback pressure gradient: A paradigm shift in physiology-guided revascularization. Cardiovasc. Revasc Med..

[53] Piroth Z., Toth G.G., Tonino P.A.L., Barbato E., Aghlmandi S., Curzen N., Rioufol G., Pijls N.H.J., Fearon W.F., Jüni P. (2017). Prognostic Value of Fractional Flow Reserve Measured Immediately After Drug-Eluting Stent Implantation. Circ. Cardiovasc. Interv..

[54] Zimbardo G., Cialdella P., DIGiusto F., Migliaro S., Anastasia G., Petrolati E., Galante D., D’Amario D., Leone A.M. (2021). Physiological assessment after percutaneous coronary intervention: The hard truth. Panminerva Med..

[55] Rimac G., Fearon W.F., De Bruyne B., Ikeno F., Matsuo H., Piroth Z., Costerousse O., Bertrand O.F. (2017). Clinical value of post-percutaneous coronary intervention fractional flow reserve value: A systematic review and meta-analysis. Am. Heart J..

[56] Diletti R., Masdjedi K., Daemen J., van Zandvoort L.J.C., Neleman T., Wilschut J., Den Dekker W.K., van Bommel R.J., Lemmert M., Kardys I. (2021). Impact of Poststenting Fractional Flow Reserve on Long-Term Clinical Outcomes: The FFR-SEARCH Study. Circ. Cardiovasc. Interv..

[57] Azzalini L., Poletti E., Demir O.M., Ancona M.B., Mangieri A., Giannini F., Carlino M., Chieffo A., Montorfano M., Colombo A. (2019). Impact of Post-Percutaneous Coronary Intervention Fractional Flow Reserve Measurement on Procedural Management and Clinical Outcomes: The REPEAT-FFR Study. J. Invasive Cardiol..

[58] Leone A.M., Migliaro S., Zimbardo G., Cialdella P., Basile E., Galante D., Di Giusto F., Anastasia G., Vicere A., Petrolati E. (2022). Safety and effectiveness of post percutaneous coronary intervention physiological assessment: Retrospective data from the post-revascularization optimization and physiological evaluation of intermediate lesions using fractional flow reserve registry. Front. Cardiovasc. Med..

[59] Piroth Z., Otsuki H., Zimmermann F.M., Ferenci T., Keulards D.C.J., Yeung A.C., Pijls N.H.J., De Bruyne B., Fearon W.F. (2022). Prognostic Value of Measuring Fractional Flow Reserve After Percutaneous Coronary Intervention in Patients with Complex Coronary Artery Disease: Insights from the FAME 3 Trial. Circ. Cardiovasc. Interv..

[60] Leone A.M. (2020). Post PCI FFR: More questions than answers?. Int. J. Cardiol..

[61] Pijls N.H.J., Kern M.J., Yock P.G., De Bruyne B. (2000). Practice and Potential Pitfalls of Coronary Pressure Measurement. Catheter. Cardiovasc. Interv..

[62] Tonino P.A., Johnson N.P. (2016). Why Is Fractional Flow Reserve After Percutaneous Coronary Intervention Not Always 1.0?. JACC Cardiovasc. Interv..

[63] van Zandvoort L.J.C., Masdjedi K., Witberg K., Ligthart J., Tovar Forero M.N., Diletti R., Lemmert M.E., Wilschut J., de Jaegere P.P.T., Boersma E. (2019). Explanation of Postprocedural Fractional Flow Reserve Below 0.85. Circ. Cardiovasc. Interv..

[64] Meneveau N., Souteyrand G., Motreff P., Caussin C., Amabile N., Ohlmann P., Morel O., Lefrançois Y., Descotes-Genon V., Silvain J. (2016). Optical Coherence Tomography to Optimize Results of Percutaneous Coronary Intervention in Patients with Non-ST-Elevation Acute Coronary Syndrome: Results of the Multicenter, Randomized DOCTORS Study (Does Optical Coherence Tomography Optimize Results of Stenting). Circulation.

[65] Ding D., Yu W., Tauzin H., De Maria G.L., Wu P., Yang F., Kotronias R.A., Terentes-Printzios D., Wolfrum M., Banning A.P. (2021). Optical flow ratio for assessing stenting result and physiological significance of residual disease. EuroIntervention.

[66] van Zandvoort L.J.C., Masdjedi K., Neleman T., Tovar Forero M.N., Wilschut J., den Dekker W., de Jaegere P.P.T., Diletti R., Zijlstra F., Van Mieghem N.M. (2020). Impact of intravascular ultrasound findings in patients with a post PCI fractional flow reserve ≤0.85 on 2 year clinical outcome. Int. J. Cardiol..

[67] Collison D. (2023). “One of These Things Is Not Like the Other”: FFR or IVUS to Guide Post-PCI Optimization?. JACC Cardiovasc. Interv..

[68] Anastasia G., Galante D., Biscaglia S., Vergallo R., Di Giusto F., Migliaro S., Petrolati E., Viceré A., Scancarello D., Marrone A. (2024). Efficacy of “Physiology-Guided PCI” Using Pressure Catheter in Comparison to Conventional Pressure Wires: A Multicenter Analysis. Am. J. Cardiol..

[69] Wolfrum M., Fahrni G., de Maria G.L., Knapp G., Curzen N., Kharbanda R.K., Fröhlich G.M., Banning A.P. (2016). Impact of impaired fractional flow reserve after coronary interventions on outcomes: A systematic review and meta-analysis. BMC Cardiovasc. Disord..

[70] Doh J.H., Nam C.W., Koo B.K., Lee S.Y., Choi H., Namgung J., Kwon S.U., Kwak J.J., Kim H.Y., Choi W.H. (2015). Clinical Relevance of Poststent Fractional Flow Reserve After Drug-Eluting Stent Implantation. J. Invasive Cardiol..

[71] Nam C.W., Rha S.W., Koo B.K., Doh J.H., Chung W.Y., Yoon M.H., Tahk S.J., Lee B.K., Lee J.B., Yoo K.D. (2011). Usefulness of coronary pressure measurement for functional evaluation of drug-eluting stent restenosis. Am. J. Cardiol..

[72] Li S.J., Ge Z., Kan J., Zhang J.J., Ye F., Kwan T.W., Santoso T., Yang S., Sheiban I., Qian X.S. (2017). Cutoff Value and Long-Term Prediction of Clinical Events by FFR Measured Immediately After Implantation of a Drug-Eluting Stent in Patients With Coronary Artery Disease: 1- to 3-Year Results From the DKCRUSH VII Registry Study. JACC Cardiovasc. Interv..

[73] Leone A.M., De Caterina A.R., Basile E., Gardi A., Laezza D., Mazzari M.A., Mongiardo R., Kharbanda R., Cuculi F., Porto I. (2013). Influence of the amount of myocardium subtended by a stenosis on fractional flow reserve. Circ. Cardiovasc. Interv..

[74] Härle T., Luz M., Meyer S., Kronberg K., Nickau B., Escaned J., Davies J., Elsässer A. (2017). Effect of Coronary Anatomy and Hydrostatic Pressure on Intracoronary Indices of Stenosis Severity. JACC Cardiovasc. Interv..

[75] Kawaguchi Y., Ito K., Kin H., Shirai Y., Okazaki A., Miyajima K., Watanabe T., Tatsuguchi M., Wakabayashi Y., Maekawa Y. (2019). Impact of Hydrostatic Pressure Variations Caused by Height Differences in Supine and Prone Positions on Fractional Flow Reserve Values in the Coronary Circulation. J. Interv. Cardiol..

[76] Zhang J., Hwang D., Yang S., Hu X., Lee J.M., Nam C.W., Shin E.S., Doh J.H., Hoshino M., Hamaya R. (2024). Angiographic Findings and Post-Percutaneous Coronary Intervention Fractional Flow Reserve. JAMA Netw. Open.

[77] Collet C., Johnson N.P., Mizukami T., Fearon W.F., Berry C., Sonck J., Collison D., Koo B.K., Meneveau N., Agarwal S.K. (2023). Impact of Post-PCI FFR Stratified by Coronary Artery. JACC Cardiovasc. Interv..

[78] Hwang D., Koo B.K., Zhang J., Park J., Yang S., Kim M., Yun J.P., Lee J.M., Nam C.W., Shin E.S. (2022). Prognostic Implications of Fractional Flow Reserve After Coronary Stenting: A Systematic Review and Meta-analysis. JAMA Netw. Open.

[79] Collison D., Didagelos M., Aetesam-Ur-Rahman M., Copt S., McDade R., McCartney P., Ford T.J., McClure J., Lindsay M., Shaukat A. (2021). Post-stenting fractional flow reserve vs coronary angiography for optimization of percutaneous coronary intervention (TARGET-FFR). Eur. Heart J..

[80] Pepine C.J. (2023). ANOCA/INOCA/MINOCA: Open artery ischemia. Am. Heart J. Plus.

[81] Jerónimo A., Paredes-Vázquez J.G., Travieso A., Shabbir A., Jiménez-Quevedo P., Macaya-Ten F., Nombela-Franco L., Núñez-Gil I.J., Salinas P., Gómez-Polo J.C. (2025). Comprehensive diagnosis in chronic coronary syndromes combining angiography and intracoronary testing: The AID-ANGIO study. EuroIntervention.

[82] Hansen B., Holtzman J.N., Juszczynski C., Khan N., Kaur G., Varma B., Gulati M. (2023). Ischemia with No Obstructive Arteries (INOCA): A Review of the Prevalence, Diagnosis and Management. Curr. Probl. Cardiol..

[83] Chioncel V., Gherasie F.A. (2024). The Role of Coronary Physiology Assessment in the Diagnosis and Treatment of Stable Angina. Dive Inside Recent Findings of Diffuse Coronary Disease Treatment. Rev. Cardiovasc. Med..

[84] Kunadian V., Chieffo A., Camici P.G., Berry C., Escaned J., Maas A.H.E.M., Prescott E., Karam N., Appelman Y., Fraccaro C. (2021). An EAPCI Expert Consensus Document on Ischaemia with Non-Obstructive Coronary Arteries in Collaboration with European Society of Cardiology Working Group on Coronary Pathophysiology & Microcirculation Endorsed by Coronary Vasomotor Disorders International Study Group. EuroIntervention.

[85] Taqueti V.R., Di Carli M.F. (2018). Coronary Microvascular Disease Pathogenic Mechanisms and Therapeutic Options: JACC State-of-the-Art Review. J. Am. Coll. Cardiol..

[86] Rizzoni D., Agabiti-Rosei C., De Ciuceis C. (2023). State of the Art Review: Vascular Remodeling in Hypertension. Am. J. Hypertens..

[87] Galante D., Viceré A., Pollio Benvenuto C., Viccaro V., Giuliana C., Todisco S., Capalbo G., Montone R., Romagnoli E., Aurigemma C. (2025). Functional assessment in angina and non-obstructive coronary arteries: From microvascular resistance reserve to subtypes of coronary microvascular dysfunction. J. Cardiovasc. Med..

[88] Maseri A., Crea F., Kaski J.C., Crake T. (1991). Mechanisms of angina pectoris in syndrome X. J. Am. Coll. Cardiol..

[89] Miner S.E.S., McCarthy M.C., Ardern C.I., Perry C.G.R., Toleva O., Nield L.E., Manlhiot C., Cantor W.J. (2023). The relationships between acetylcholine-induced chest pain, objective measures of coronary vascular function and symptom status. Front. Cardiovasc. Med..

[90] Lanza G.A., Careri G., Crea F. (2011). Mechanisms of coronary artery spasm. Circulation.

[91] Zimmermann F.M., Pollio Benvenuto C. (2025). Bringing order to chaos. Invasive functional assessment for all patients in the cath lab?. EuroIntervention.

[92] Ong P., Camici P.G., Beltrame J.F., Crea F., Shimokawa H., Sechtem U., Kaski J.C., Bairey Merz C.N. (2018). Coronary Vasomotion Disorders International Study Group (COVADIS). International standardization of diagnostic criteria for microvascular angina. Int. J. Cardiol..

[93] Scarsini R., Campo G., DISerafino L., Zanon S., Rubino F., Monizzi G., Biscaglia S., Ancona M., Polimeni A., Niccoli G. (2023). #FullPhysiology: A systematic step-by-step guide to implement intracoronary physiology in daily practice. Minerva Cardiol. Angiol..

[94] Leone A.M., Galante D., Viceré A., Marrone A., Verardi F.M., Giuliana C., Pollio Benvenuto C., Viccaro V., Todisco S., Erriquez A. (2025). Functional coronary assessment in angina with intermediate coronarystenosis: The #FullPhysiology approach. Eur. Heart J..

[95] Ford T.J., Stanley B., Good R., Rocchiccioli P., McEntegart M., Watkins S., Eteiba H., Shaukat A., Lindsay M., Robertson K. (2018). Stratified Medical Therapy Using Invasive Coronary Function Testing in Angina: The CorMicA Trial. J. Am. Coll. Cardiol..

[96] Buono A., Pedrotti P., Soriano F., Veas N., Oliva F., Oreglia J., Ammirati E. (2019). L’infarto miocardico senza ostruzione coronarica significativa (MINOCA): Inquadramento diagnostico, patogenesi, terapia e prognosi. G. Ital. Cardiol..

[97] Petrolati E., Montone R.A., Leone A.M., Ricchiuto A., La Vecchia G., Rinaldi R., Fracassi F., Romagnoli E., Paraggio L., Burzotta F. (2023). L’infarto miocardico in assenza di coronaropatia ostruttiva: Work-up diagnostic nel laboratorio di Emodinamica. G. Ital. Cardiol..

[98] Gurgoglione F.L., Vignali L., Montone R.A., Rinaldi R., Benatti G., Solinas E., Leone A.M., Galante D., Campo G., Biscaglia S. (2024). Coronary Spasm Testing with Acetylcholine: A Powerful Tool for a Personalized Therapy of Coronary Vasomotor Disorders. Life.

[99] Montone R.A., Rinaldi R., Del Buono M.G., Gurgoglione F., La Vecchia G., Russo M., Caffè A., Burzotta F., Leone A.M., Romagnoli E. (2022). Safety and prognostic relevance of acetylcholine testing in patients with stable myocardial ischaemia or myocardial infarction and non-obstructive coronary arteries. EuroIntervention.

[100] Montone R.A., Cosentino N., Gorla R., Biscaglia S., La Vecchia G., Rinaldi R., Caffè A., Resta M., Erriquez A., Bedogni F. (2025). Stratified treatment of myocardial infarction with non-obstructive coronary arteries: The PROMISE trial. Eur. Heart J..

[101] Reynolds H.R., Maehara A., Kwong R.Y., Sedlak T., Saw J., Smilowitz N.R., Mahmud E., Wei J., Marzo K., Matsumura M. (2021). Coronary Optical Coherence Tomography and Cardiac Magnetic Resonance Imaging to Determine Underlying Causes of Myocardial Infarction with Nonobstructive Coronary Arteries in Women. Circulation.

[102] Remmelink M., Sjauw K.D., Yong Z.Y., Haeck J.D., Vis M.M., Koch K.T., Tijssen J.G., de Winter R.J., Henriques J.P., Piek J.J. (2013). Coronary microcirculatory dysfunction is associated with left ventricular dysfunction during follow-up after STEMI. Neth. Heart J..

[103] Galli M., Niccoli G., De Maria G., Brugaletta S., Montone R.A., Vergallo R., Benenati S., Magnani G., D’Amario D., Porto I. (2024). Coronary microvascular obstruction and dysfunction in patients with acute myocardial infarction. Nat. Rev. Cardiol..

[104] De Maria G.L., Alkhalil M., Wolfrum M., Fahrni G., Borlotti A., Gaughran L., Dawkins S., Langrish J.P., Lucking A.J., Choudhury R.P. (2019). Index of Microcirculatory Resistance as a Tool to Characterize Microvascular Obstruction and to Predict Infarct Size Regression in Patients with STEMI Undergoing Primary PCI. JACC Cardiovasc. Imaging.

[105] Fearon W.F., Shah M., Ng M., Brinton T., Wilson A., Tremmel J.A., Schnittger I., Lee D.P., Vagelos R.H., Fitzgerald P.J. (2008). Predictive value of the index of microcirculatory resistance in patients with ST-segment elevation myocardial infarction. J. Am. Coll. Cardiol..

[106] Fahrni G., Wolfrum M., De Maria G.L., Cuculi F., Dawkins S., Alkhalil M., Patel N., Forfar J.C., Prendergast B.D., Choudhury R.P. (2017). Index of Microcirculatory Resistance at the Time of Primary Percutaneous Coronary Intervention Predicts Early Cardiac Complications: Insights From the OxAMI (Oxford Study in Acute Myocardial Infarction) Cohort. J. Am. Heart Assoc..

[107] Scarsini R., Shanmuganathan M., De Maria G.L., Borlotti A., Kotronias R.A., Burrage M.K., Terentes-Printzios D., Langrish J., Lucking A., Fahrni G. (2021). OxAMI Study Investigators. Coronary Microvascular Dysfunction Assessed by Pressure Wire and CMR After STEMI Predicts Long-Term Outcomes. JACC Cardiovasc. Imaging.

[108] El Farissi M., Zimmermann F.M., De Maria G.L., van Royen N., van Leeuwen M.A.H., Carrick D., Carberry J., Wijnbergen I.F., Konijnenberg L.S.F., Hoole S.P. (2023). The Index of Microcirculatory Resistance After Primary PCI: A Pooled Analysis of Individual Patient Data. JACC Cardiovasc. Interv..

[109] Scarsini R., Shanmuganathan M., Kotronias R.A., Terentes-Printzios D., Borlotti A., Langrish J.P., Lucking A.J., Ribichini F., Ferreira V.M., OxAMI Study Investigators (2021). Angiography-derived index of microcirculatory resistance (IMRangio) as a novel pressure-wire-free tool to assess coronary microvascular dysfunction in acute coronary syndromes and stable coronary artery disease. Int. J. Cardiovasc. Imaging.

[110] De Maria G.L., Scarsini R., Shanmuganathan M., Kotronias R.A., Terentes-Printzios D., Borlotti A., Langrish J.P., Lucking A.J., Choudhury R.P., Kharbanda R. (2020). Angiography-derived index of microcirculatory resistance as a novel, pressure-wire-free tool to assess coronary microcirculation in ST elevation myocardial infarction. Int. J. Cardiovasc. Imaging.

[111] Scarsini R., Kotronias R.A., Della Mora F., Portolan L., Andreaggi S., Benenati S., Marin F., Sgreva S., Comuzzi A., Butturini C. (2024). Angiography-Derived Index of Microcirculatory Resistance to Define the Risk of Early Discharge in STEMI. Circ. Cardiovasc. Interv..

[112] Pollio Benvenuto C., Galante D., Zimmermann F., Viceré A., Viccaro V., Giuliana C., Todisco S., Bellamoli M., Bettari L., Maffeo D. (2025). Angiography-Derived Index of Microvascular Resistance in Patients with Anterior ST-Segment Elevation Myocardial Infarction After Successful Primary Percutaneous Coronary Intervention. Am. J. Cardiol..

